# Survival Analysis of Irish Amyotrophic Lateral Sclerosis Patients Diagnosed from 1995–2010

**DOI:** 10.1371/journal.pone.0074733

**Published:** 2013-09-30

**Authors:** James Rooney, Susan Byrne, Mark Heverin, Bernie Corr, Marwa Elamin, Anthony Staines, Ben Goldacre, Orla Hardiman

**Affiliations:** 1 Academic Unit of Neurology, Trinity Biomedical Sciences Institute, Dublin, Ireland; 2 Department of Neurology, Beaumont Hospital, Dublin, Ireland; 3 School of Nursing and Human Sciences, Dublin City University, Dublin, Ireland; 4 Department of Epidemiology, London School of Hygiene and Tropical Medicine, London, United Kingdom; Genentech, United States of America

## Abstract

**Introduction:**

The Irish ALS register is a valuable resource for examining survival factors in Irish ALS patients. Cox regression has become the default tool for survival analysis, but recently new classes of flexible parametric survival analysis tools known as Royston-Parmar models have become available.

**Methods:**

We employed Cox proportional hazards and Royston-Parmar flexible parametric modeling to examine factors affecting survival in Irish ALS patients. We further examined the effect of choice of timescale on Cox models and the proportional hazards assumption, and extended both Cox and Royston-Parmar models with time varying components.

**Results:**

On comparison of models we chose a Royston-Parmar proportional hazards model without time varying covariates as the best fit. Using this model we confirmed the association of known survival markers in ALS including age at diagnosis (Hazard Ratio (HR) 1.34 per 10 year increase; 95% CI 1.26–1.42), diagnostic delay (HR 0.96 per 12 weeks delay; 95% CI 0.94–0.97), Definite ALS (HR 1.47 95% CI 1.17–1.84), bulbar onset disease (HR 1.58 95% CI 1.33–1.87), riluzole use (HR 0.72 95% CI 0.61–0.85) and attendance at an ALS clinic (HR 0.74 95% CI 0.64–0.86).

**Discussion:**

Our analysis explored the strengths and weaknesses of Cox proportional hazard and Royston-Parmar flexible parametric methods. By including time varying components we were able to gain deeper understanding of the dataset. Variation in survival between time periods appears to be due to missing data in the first time period. The use of age as timescale to account for confounding by age resolved breaches of the proportional hazards assumption, but in doing so may have obscured deficiencies in the data. Our study demonstrates the need to test for, and fully explore, breaches of the Cox proportional hazards assumption. Royston-Parmar flexible parametric modeling proved a powerful method for achieving this.

## Introduction

Progress in determining the cause or causes of Amyotrophic Lateral Sclerosis (ALS) and the development of effective treatments has been remarkably slow. With an incidence in Europe of 2–3 people per 100,000 of population [1], large nationalized disease registries such as the Irish ALS register [2] prospectively collecting patients over a long period are necessary to fully appreciate the diverse clinical features of the condition.

A systematic review of ALS survival found that both bulbar onset disease and El-Escorial criteria *definite* disease have been associated with a significantly poorer prognosis [3]. Diagnostic delay is a consistently important survival factor with a longer time between symptom onset and diagnosis being associated with improved survival in many studies [3]. Age at onset is also a strong adverse prognostic factor in ALS in the majority of studies [3], whilst gender is not (despite a higher rate of bulbar disease in women) [3]. A recent prospective study from our group has found that the presence of executive dysfunction (HR 3.44 95% CI: 1.45–8.18) or fronto-temporal dementia (FTD) (HR 2.67 95% CI: 1.04–6.85) in incident Irish ALS patients is significantly associated with a poorer prognosis [4].

The sole drug licensed for ALS is the drug riluzole. In 2012 a Cochrane meta-analysis of riluzole for ALS, found that 100 mg of riluzole daily was associated with HR of 0.84 (95% CI: 0.698–0.997) [5]. Attendance at a specialist multidisciplinary clinic also improves survival [1], [3], [6]. A randomised controlled trial of non-invasive ventilation (NIV) in ALS patients found a median survival benefit of 205 days (P = 0.006) in those using NIV versus controls [7]. There has also been interest in gastrostomy as a therapy due to the importance of maintaining nutritional status [3], however the benefit of this in terms of survival remains unclear [1].

The Cox Proportional Hazards (PH) model [8], which has become the most common modeling tool for survival analysis [9], is considered a semi-parametric method [10]. That is, the Cox model makes no assumptions about the shape of the hazard over time, but instead assumes that the ratio of the hazards between categories remains constant over time [9], [10]. This offers advantages over fully parametric models which require that assumptions are made about the distribution of the underlying hazard which can be quite restrictive [9], [10]. Unfortunately in doing this, information regarding the baseline hazard that may be of interest is lost [9]. Difficulties also arise when the PH assumption is breached. However Cox models can be extended to allow specified variables to have hazard ratios that vary over time (this is achieved by splitting the analysis time and calculating hazard ratios for each time period) - such variables are known as time varying covariates (TVC’s) [9], [10], [11]. Finally, in prediction models, it is not clear how Cox PH models can be validated in external populations as the Cox method leads to an overfitting of the baseline hazard [9], [12]. Cox models have been extensively used to study survival in ALS [4], [5], [6], [13], [14], [15], [16], [17].

As a further development, the use of age at diagnosis as the timescale in Cox models has emerged as a popular technique for taking account of confounding by age due to the fact that such confounding is often poorly controlled for using conventional methods [18]–[20]. Under such a model, study entry occurs at date of birth - the effect of age being taken into account by the non-parametric aspect of the Cox model – thus providing a superior correction for the effect of age over conventional methods [19]. Consequently, this benefit comes at the penalty of being unable to quantify the association of age itself with survival.

In 2002, motivated by the limitations of Cox models, Royston & Parmar proposed a new family of flexible parametric survival models [12]. These models make use of the Aranda-Ordaz family of link functions to estimate hazard functions flexibly, including the proportional hazards, proportional odds and probit-scale models as special cases [9], [12]. Through the use of restricted cubic splines to model the baseline hazard, greater flexibility is added, so extending the range of hazard functions which can be modeled [9], [12]. Finally, time varying components can be estimated with a varying number of cubic spline knots [9], [12].

The current study aims to model the survival of Irish ALS patients diagnosed between 1^st^ of January 1995 and 31^st^ of December 2010. On initial analysis using Cox PH regression, it quickly became apparent that there are breaches of the PH assumptions that are not adequately addressed by Cox based methods. Therefore we extend our analysis to include Royston-Parmar (RP) flexible parametric modeling with the aim of comparing RP modeling to Cox PH, and also to further explore the breaches of the PH assumption.

## Methods

### Data Sources

The Irish ALS register was established in 1995 to follow incident ALS patients over time using multiple independent data sources and capture-recapture methodology [2]. This analysis includes data on patients diagnosed between 1^st^ of January 1995 and 31^st^ December 2010 from the register, augmented with data on riluzole prescription obtained from the Health Services Executive of Ireland (HSE), data on the prescription of NIV obtained from the NIV systems supplier, and gastrostomy insertion data from hospital records to compile our final data set. Explanatory variables in the final dataset included sex, age at diagnosis, year of diagnosis, diagnostic delay, El-Escorial category, site of disease onset, familial disease, attendance at specialist ALS clinic, history of riluzole prescription, NIV use, RIG (radiographically inserted gastrostomy) insertion and PEG (percutaneously inserted gastrostomy) insertion. All interventions were coded on an intention-to-treat basis and attendance at specialist ALS clinic was coded as yes for anyone with at least one visit.

### Ethics Statement

The Irish ALS Register complies with Irish Data protection legislation (1988 and 2003), and has been approved by Beaumont Hospital Ethics Committee (02/28 and 05/49). Approval for the study is from Beaumont hospital ethics committee (05/49). Verbal consent is obtained from all participants for inclusion on the Irish ALS Register. All cases have written documentation of verbal approval. The Irish Data Protection Commissioner has provided written confirmation of compliance with Irish data protection legislation. This approval is on file with the local IRB. On consideration of the privacy of individual patients it was decided that results from categories with less than 5 entries will be omitted.

### Software

Data was imported into Stata version 11 [21] for analysis. Standard Stata commands (*stci*, *strate*, *stmh* & *stcox*) were used for the classical and Cox PH analysis, whilst the additional Stata command *stpm2* for implementing RP methods was downloaded from the Statistical Software Components archive [22].

### Analysis Strategy

After consideration of the appropriate timescale, the date of diagnosis was used to mark study entry as opposed to date of symptom onset. Whilst date of onset may most appropriately reflect the natural timescale of ALS, the date of onset is subject to recall bias. Furthermore, since diagnostic delay is considered prognostic, if date of onset is used to mark study entry and diagnostic delay is used as a predictive variable then there is potential for correlation between diagnostic delay and survival time, as diagnostic delay forms a large fraction of overall survival time. A third consideration is that the hazard associated with interventions, e.g. riluzole, only exists after interventions are made, i.e. after diagnosis. Therefore in survival models including terms for interventions the use of date of diagnosis as study entry better reflects the effect on survival associated with given interventions than does the date of onset. As we were interested in the hazard ratios associated with interventions in our analysis, time since diagnosis was used as timescale for all analyses, except in specified models where age at diagnosis was used to explore the effect on breaches of the PH assumption. (However, to illustrate the difference between date of onset and date of diagnosis timescales the final preferred model was constructed for both timescales – this data is presented in the table S1).

Initially, descriptive statistics were estimated and the Nelson-Aalen cumulative hazard for the overall cohort was graphed. Non-parametric methods were used to determine crude survival rates for each stratum of each explanatory variable.

#### Cox proportional hazards regression

Stata’s *stcox* command was used to perform Cox PH regression. Entries with missing values were dropped prior to modeling. Initially all variables were included, with variables sequentially removed from the model via manually implemented backwards elimination. When compared to forward selection, backwards elimination is more likely to detect significant variables when they are subject to suppressor effects of associated variables and is therefore preferred by numerous authors [9], [23], [24]. Successive models were compared using likelihood ratio testing (LRT) with P<0.05 as the significance threshold. Next, all pairwise combinations of interaction terms were tested and compared to the base model via LRT. The model with lowest P value after comparison via LRT was selected as the best-fit model. Formal testing of the PH assumption was performed using the Stata command *estat phtest*, and scaled Schoenfeld residuals were generated for graphical analysis of the PH assumption. To correct for residual confounding by age, the timescale was reset to use age as follow up time and the model recalculated omitting age [18]–[20].

#### Cox regression with time varying covariates

To allow for breaches of the PH assumption the best-fit model was extended by allowing for time varying covariates (TVC’s) for those variables that were in breach of the PH assumption. Forward selection was used to select variables to include time varying covariates as suggested by Royston and Lambert for including TVC’s in RP models [9]. As this involved a large number of comparisons a significance threshold of P<0.001 was used. Hazard ratios for TVC’s were plotted versus time. This was implemented using the *tvc* option of Stata’s *stcox* command.

#### Royston-parmar propotional hazards models

The *stpm2* command was used to build RP-PH models. It should be noted that Royston-Parmar methods are capable of utilizing proportional odds and probit assumptions as an alternative to proportional hazards - the choice of model typically being data-driven, however given our aim of exploring breaches of the Cox PH assumption we restricted the RP modeling to use the proportional hazards assumption. The Akaike Information Criterion (AIC) and Bayesian Information Criterion (BIC) were used to select the optimum number of spline knots [9]. It was determined that 2 internal spline knots were optimum (meaning d.f. = 3 for the *stpm2* command). Similar to Cox PH model building, backwards elimination and LRT were used to model-build with P<0.05 as the significance threshold. Again all pairwise combinations of interaction terms were tested to arrive at a final best-fit model. As Schoenfeld residuals are not available from the *stpm2* command this step was omitted for RP-PH models. Instead, all variables were examined for interaction with time by allowing for TVC’s. Forward selection was used to add TVC’s to the best-fit RP-PH model with P<0.001 as threshold for significance. Again, the AIC and BIC were used to determine the optimum degrees of freedom (i.e. number of spline knots) of each time dependent variable [9].

## Results

### Descriptive Statistics

In total 1,282 incident cases were included. Of these, only 2 had unknown vital status at the end of follow-up, and only 1 other was known to have died but the precise date was unknown, therefore loss to follow-up was minimal. Table 1a displays summary statistics for the data set including numbers of missing values. Table 1b displays the summary statistics of all observations excluded due to missing values per category of each variable.

**Table 1:** Descriptive statistics of Irish ALS cases from 1^st^ Jan 1995 to 31^st^ Dec 2010.

a) Complete Cohort (n = 1,282)					b) Distribution of cases excluded due to missing values (n = 196)	
Variable	n	Strata	Num per stratum/Mean (std dev)	Num missing	Num per stratum/Mean (std dev)	P value*
ID	1,282	–	–	0	–	–
Age at diagnosis	1,276	–	64.6 (11.8)	6	66.3 (11.6)	**0.0336**
Date of onset	1,118	–	–	159	–	–
Date of diagnosis	1,282	–	–	0	–	–
Year of Dx	1,282	1995–2000	428 (33.4%)	0	143 (73.0%)	
		2001–2005	408 (31.8%)		32 (16.3%)	
		2006–2010	446 (34.8%)		21 (10.7%)	**<0.001**
Sex	1,282	Male	723 (56.4%)	0	106 (54.1%)	
		Female	559 (43.6%)		90 (45.9%)	0.482
El Escorial category	1,262	Definite	709 (56.2%)	20	114 (64.7%)	
		Probable	386 (30.6%)		52 (29.6%)	
		Possible	153 (12.1%)		9 (5.1%)	
		Suspected	14 (1.1%)		1 (0.6%)	**0.005**
Site of onset	1,272	Limb	743 (58.4%)	9	91 (48.7%)	
		Bulbar	465 (36.5%)		83 (44.4%)	
		Generalised	65 (5.1%)		13 (6.9%)	**0.012**
Familial	1,282	Sporadic	1,202 (93.8%)	0	189 (96.4%)	
		Familial	80 (6.2%)		7 (3.6%)	0.058
Attended ALS clinic	1,276	No	462 (36.2%)	6	83 (43.7%)	
		Yes	814 (63.8%)		107 (56.3%)	**0.022**
Prescribed Riluzole	1,271	No	412 (32.4%)	11	121 (65.4%)	
		Yes	859 (67.6%)		64 (34.6%)	**<0.001**
RIG insertion	1,269	No	1,126 (88.7%)	13	179 (97.8%)	
		Yes	143 (11.3%)		4 (2.2%)	**<0.001**
PEG insertion	1,269	No	1,083 (85.3%)	13	153 (83.6%)	
		Yes	186 (14.7%)		30 (16.4%)	0.498
Prescribed NIV	1,264	No	983 (77.8%)	18	170 (95.5%)	
		Yes	281 (22.2%)		8 (4.5%)	**<0.001**

* P value for age at diagnosis obtained from t-test of those included vs those excluded. P value for all other tests calculated using Fishers exact test of those included vs those excluded.

Of the complete cohort, 56.4% of all cases were male. 58.4% of cases had limb onset, whilst 36.5% of cases had bulbar onset with the remainder having more generalized onset. Analysis of site of onset by gender revealed that 56.6% of bulbar onset cases were female whilst 64.6% of limb onset cases were male. 56.2% of cases were in the definite El Escorial category, with 30.6% probable, 12.1% possible and 1.1% suspected. There were 196 (15%) cases with missing values in one or more fields – these were included in non-parametric analysis but excluded from Cox PH and Royston-Parmar analyses. Those excluded had significant associations with grouped year of diagnosis, riluzole prescription, RIG insertion, NIV prescription and less significant associations with age at diagnosis, El Escorial category, site of onset and ALS clinic attendance.

### Nonparametric Analysis

**Figure 1:**
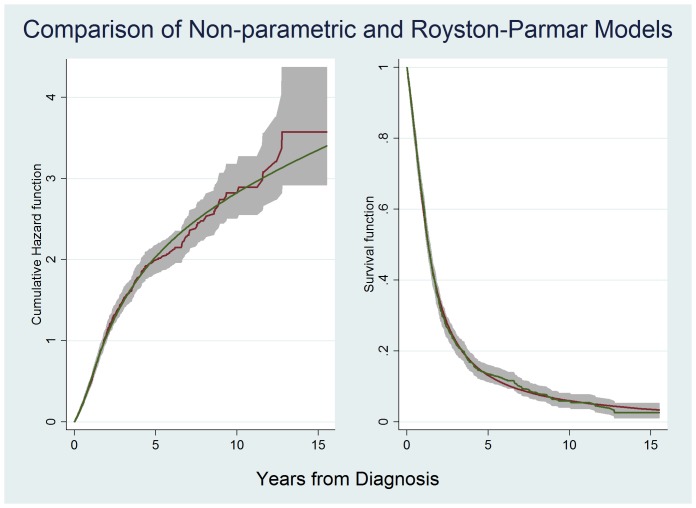
Cumulative Hazard function and Survival function of Non-parametric and Royston-Parmar models. The graph on the left shows cumulative hazards estimated using the Nelson-Aalen method (red line) with 95% CI’s (grey area) and the cumulative hazard estimated by Royston-Parmar PH (df 3) modeling (green line). The graph on the right shows the survival function estimated using the Kaplan-Meier method with 95% CI’s (grey area) and the survival function estimated by Royston-Parmar PH (df 3) modeling (green line). These graphs were based on the full cohort minus those missing data for key variables (n = 1086). As can be seen the Royston-Parmar models provides excellent fit when compared to non-parametric methods.

Results of non-parametric survival analysis are shown in table 2. Median survival from symptom onset was 2.39 years while median survival from date of diagnosis was 1.27 years. Factors significantly associated with worsened survival included female gender (HR 1.29; 95% CI 1.14–1.46), increasing age group, bulbar onset (HR 1.94; 95% CI 1.71–2.21), the definite El-Escorial category (HR 1.88; 95% CI 1.53–2.31), the probable El-Escorial category (HR 1.48; 95% CI 1.19–1.84) and PEG insertion (HR 1.20; 95% CI 1.02–1.42). Protective factors included familial disease (HR 0.74; 95% CI 0.57–0.96), increasing diagnostic delay, attendance at the ALS clinic (HR 0.65; 95% CI 0.58–0.74) and history of riluzole prescription (HR 0.65; 95% CI 0.57–0.74). RIG insertion and NIV use had no significant effect on crude analysis. The non-parametric analysis shows, surprisingly, an apparent rise in HR over the years of diagnosis with 2006–2010 showing increased hazard (HR1.26; 95% CI 1.08–1.46) when compared to 1995–2000. The cumulative hazard function and survival functions for the cohort minus those with missing key variables are shown in Figure 1.

**Table 2:** Survival times and crude death rates for incident Irish ALS patients from symptom onset.

Risk Factor (n)	Category	No. incategory	No. died	Person-years	MedianSurvival (yrs)	Rate (95% CI) per person year	Rate Ratio	P value
Sex (1277)	Male	719	579	1,437	1.31	0.40 (0.37–0.44)	–	
	Female	558	473	909	1.24	0.52 (0.48–0.57)	1.29 (1.14–1.46)	**<0.0001**
Age group at Dx‡ (1,271)	<25 yrs	o	o	o	o	o	o	ns
	25–44	75	48	263	3.20	0.18 (0.14–0.24)	0.32 (0.23–0.43)	**<0.0001**
	45–54	174	137	445	1.88	0.31 (0.26–0.36)	0.54 (0.44–0.65)	**<0.0001**
	55–64	353	285	719	1.52	0.40 (0.35–0.45)	0.69 (0.59–0.80)	**<0.0001**
	65–74	425	359	623	1.05	0.58 (0.52–0.64)	–	–
	>75 yrs	240	216	280	0.72	0.77 (0.68–0.88)	1.34 (1.13–1.59)	**0.0006**
Year of Dx (1,277)	1995–2000	427	412	994	1.42	0.41 (0.38–0.46)	–	**–**
	2001–2005	405	362	818	1.20	0.44 (0.40–0.49)	1.07 (0.93–1.23)	0.3617
	2006–2010	445	278	534	1.22	0.52 (0.46–0.59)	1.26 (1.08–1.46)	**0.0033**
El-Escorial category* (1,259)	Suspected	14	10	43	2.85	0.23 (0.12–0.43)	0.82 (0.43–1.56)	0.5435
	Possible	153	108	380	2.06	0.28 (0.24–0.34)	–	–
	Probable	385	326	776	1.44	0.42 (0.38–0.47	1.48 (1.19–1.84)	**0.0004**
	Definite	707	593	1,110	1.16	0.53 (0.49–0.58)	1.88 (1.53–2.31)	**<0.0001**
Site of onset (1,268)	Limb	742	581	1,608	1.57	0.36 (0.33–0.39)	–	–
	Bulbar	462	406	578	1.07	0.70 (0.64–0.77)	1.94 (1.71–2.21)	**<0.0001**
	General	64	57	150	0.88	0.38 (0.29–0.49)	1.05 (0.80–1.38)	0.7205
Familial ALS ? (1,277)	Sporadic	1197	995	2,177	1.25	0.46 (0.43–0.49)	–	**–**
	Familial	80	57	169	1.58	0.34 (0.26–0.44)	0.74 (0.57–0.96)	**0.0255**
Diagnostic Delay (1,118)	<31 weeks	380	321	584	1.14	0.55 (0.49–0.61)	–	–
	31–55 wks	369	295	646	1.33	0.46 (0.41–0.51)	0.83 (0.71–0.98)	**0.0225**
	>55 weeks	369	279	875	1.54	0.32 (0.28–0.36)	0.58 (0.50–0.68)	**<0.0001**
Attended ALS clinic (1,271)	No	459	414	696	0.93	0.60 (0.54–0.66)	–	**–**
	Yes	812	634	1,637	1.51	0.39 (0.36–0.42)	0.65 (0.58–0.74)	**<0.0001**
Prescribed riluzole (1,267)	No	410	374	608	0.85	0.61 (0.56–0.68)	–	**–**
	Yes	857	676	1,696	1.46	0.40 (0.37–0.43)	0.65 (0.57–0.74)	**<0.0001**
RIG inserted (1,265)	No	1122	938	2,019	1.25	0.46 (0.44–0.50)	–	–
	Yes	143	110	282	1.54	0.39 (0.32–0.47)	0.84 (0.69–1.02)	0.0844
PEG inserted (1,265)	No	1079	875	1,976	1.31	0.44 (0.41–0.47)	–	–
	Yes	186	173	324	1.13	0.53 (0.46–0.62)	1.20 (1.02–1.42)	**0.0252**
Prescribed NIV (1,260)	No	980	810	1,774	1.26	0.46 (0.43–0.49)	–	–
	Yes	280	235	513	1.27	0.46 (0.40–0.52)	1.00 (0.87–1.16)	0.9646

* Possible ALS used as baseline category for El-Escorial category rate ratio calculations.

‡ 65–74 is peak age group and is used as the baseline category for rate ratio calculations.

Dx = diagnosis; o = counts less than 5 omitted; ns = not significant.

Overall n = 1,282. Overall median survival from diagnosis = 1.27 yrs (95% CI:1.20–1.36).

Overall median survival from symptom onset = 2.39years (95% CI: 2.26–2.54).

### Cox Regression Models (No TVC’s)

The results of Cox PH models are shown in table 3. Model building led to inclusion of age at diagnosis and diagnostic delay as linear effects, period of diagnosis, site of onset, El-Escorial category, attendance of ALS clinic, riluzole and NIV prescription in the best-fit models, with an interaction found between site of onset and NIV prescription. The Cox PH model (model 1) globally failed the PH assumption (P<0.0001). Individual variables/strata failing the PH assumption included riluzole prescription (P<0.0001), diagnosis between 2001 & 2005 (P = 0.0001), diagnosis between 2006 & 2010 (P = 0.0180) and attendance at ALS clinic (P = 0.0044). Using age as timescale (model 2), no variables failed the PH assumption. On the new timescale the majority of HR’s changed little, however the HR for El Escorial categories increased, and diagnosis between 2006–2010 was associated with a significantly higher HR.

**Table 3:** Hazard ratios from Cox Proportional Hazards models.

Model	Cox PH		Cox PH		Royston-Parmar PH (df 3)		Royston-Parmar − PH(df 3)+TVC’s	
Model Number	1		2		3		4	
Timescale	Time on study		Age		Time on study		Time on study	
Variable	HR (95% CI)	P values	HR (95% CI)	P values	HR (95% CI)	P values	HR (95% CI)	P values
Strata								
**Age dx** (per 10 yrs increase)	1.34 (1.26–1.43)	**<0.001**	–	–	1.34 (1.26–1.42)	**<0.001**	1.34 (1.26–1.43)	**<0.001**
**El Escorial**								
Possible	1	–	1	–	1	–	1	–
Suspected	0.96 (0.50–1.86	0.914	1.12 (0.57–2.15)	0.763	0.96 (0.50–1.84)	0.901	0.98 (0.51–1.88)	0.949
Probable	1.31 (1.04–1.65)	**0.021**	1.32 (1.05–1.67)	**0.019**	1.30 (1.03–1.64)	**0.026**	1.31 (1.04–1.65)	**0.023**
Definite	1.48 (1.19–1.86)	**0.001**	1.55 (1.24–1.95)	**<0.001**	1.47 (1.17–1.84)	**0.001**	1.52 (1.22–1.91)	**<0.001**
**Site of onset** (no NIV)								
Limb onset	1	–	1	–	1	–	1	–
Bulbar onset	1.60 (1.35–1.89)	**<0.001**	1.60 (1.35–1.90)	**<0.001**	1.58 (1.33–1.87)	<0.001	1.54 (1.30–1.83)	**<0.001**
General onset	1.15 (0.82–1.63)	0.411	1.17 (0.83–1.66)	0.372	1.17 (0.83–1.65)	0.371	1.14 (0.80–1.60)	0.470
**Dx Delay** (per 12 weeks delay)	0.96 (0.94–0.97)	**<0.001**	0.95 (0.94–0.97)	**<0.001**	0.96 (0.94–0.97)	**<0.001**	0.96 (0.94–0.97)	**<0.001**
**Year Dx**								
1995–2000	1	–	1	–	1	–	TVC	TVC
2001–2005	1.29 (1.08–1.54)	**0.005**	1.30 (1.09–1.55)	**0.004**	1.26 (1.06–1.51)	**0.009**	1	–
2006–2010	1.16 (0.96–1.40)	0.127	1.25 (1.04–1.50)	**0.020**	1.15 (0.96–1.39)	0.138	o	o
**Attend ALS clinic**	0.74 (0.64–0.86)	**<0.001**	0.77 (0.66–0.89)	**0.001**	0.74 (0.64–0.86)	**<0.001**	0.73 (0.63–0.85)	**<0.001**
**Riluzole**	0.72 (0.61–0.85)	**<0.001**	0.74 (0.63–0.88)	**<0.001**	0.72 (0.61–0.85)	**<0.001**	TVC	TVC
**NIVusage**								
NIV+Limb onset	1.51 (1.23–1.86)	**<0.001**	1.40 (1.13–1.72)	**0.002**	1.49 (1.21–1.84)	**<0.001**	1.47 (1.20–1.81)	**<0.001**
NIV+Bulbar onset	1.08 (0.81–1.42)	0.601	1.15 (0.87–1.53)	0.320	1.09 (0.83–1.44)	0.536	1.05 (0.79–1.38)	0.753
NIV+General onset	2.80 (1.34–5.85)	**0.006**	2.50 (1.18–5.28)	**0.017**	2.74 (1.31–5.71)	**0.007**	2.26 (1.08–4.71)	**0.030**

Note that constant terms output from the Royston-Parmar have not been displayed.

P values are from Wald test of variables in regression outputs.

TVC – indicates time varying covariates which are graphically displayed in Figure 3.

o – implies omitted from model by Stata due to colinearity. This occurred as the *stpm2* command does not recognize factor variables and dummy variables were used.

In Cox models, underlined terms imply failure of Cox PH assumption using Stata command *estat phtest, detail*. No variable failed Cox PH assumption i.

### Royston-Parmar Models (no TVC’s)

Model building under the RP-PH framework led to selection of the same variables and interaction terms in the best-fit Cox model. Model results are shown in table 3. In general the hazard ratios were in very close agreement between Cox and RP models with the time on study timescale (model 1 & 3). When an RP model was fitted with age as timescale (data not shown), the majority of HR’s were in agreement with the Cox model (model 2).

Through Royston-Parmar modeling (model 3) we found that each 10-year increase in age was associated with poorer prognosis (HR 1.34 95% CI: 1.26–1.42). Each 12-week delay in diagnosis was associated with improved prognosis (HR 0.96 95% CI: 0.94–0.97). Bulbar onset disease was associated with significantly poorer prognosis (HR 1.58 95% CI: 1.33–1.87) as was the Definite El-Escorial category (HR 1.47 95% CI: 1.17–1.84). Attendance at a specialist ALS clinic (HR 0.74 95% CI: 0.64–0.86) and riluzole prescription (HR 0.72 95% CI: 0.61–0.85) were both associated with improved prognosis. Model 3 also found a significantly poorer survival for the years 2001–2005 (HR 1.26 95% CI: 1.06–1.51) compared to the years 1995–2000.

**Figure 2:**
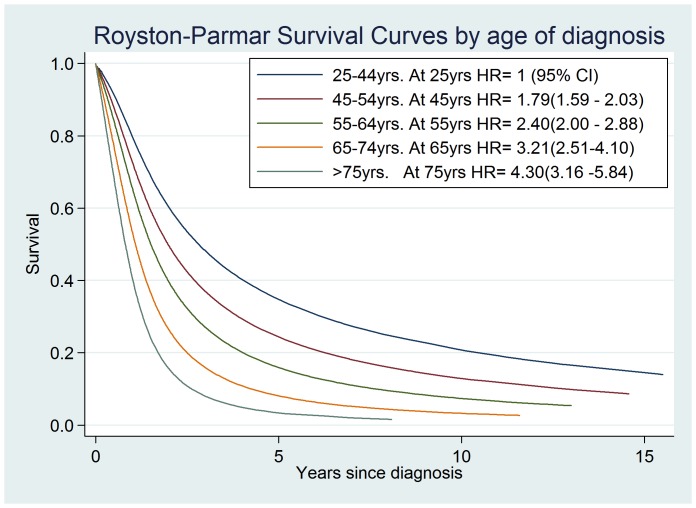
Royston-Parmar Survival Curves by diagnostic age group. Predicted cumulative survival curves based on model 3. Curves represent mean survival for each age group. The legend contains hazard ratios with 95% CI’s for specific ages determined from model parameters and taking 25 yrs to represent the baseline age risk.

As RP modeling provides a smoothed estimate of baseline hazard, this allowed us to calculate adjusted survival curves for subgroups of the population. Survival curves for different age groups based on RP regression (model 3) are shown in Figure 2, where it can be seen that survival decreases significantly with increasing age group.

### Cox PH and RP-PH Models with TVC’s

**Figure 3:**
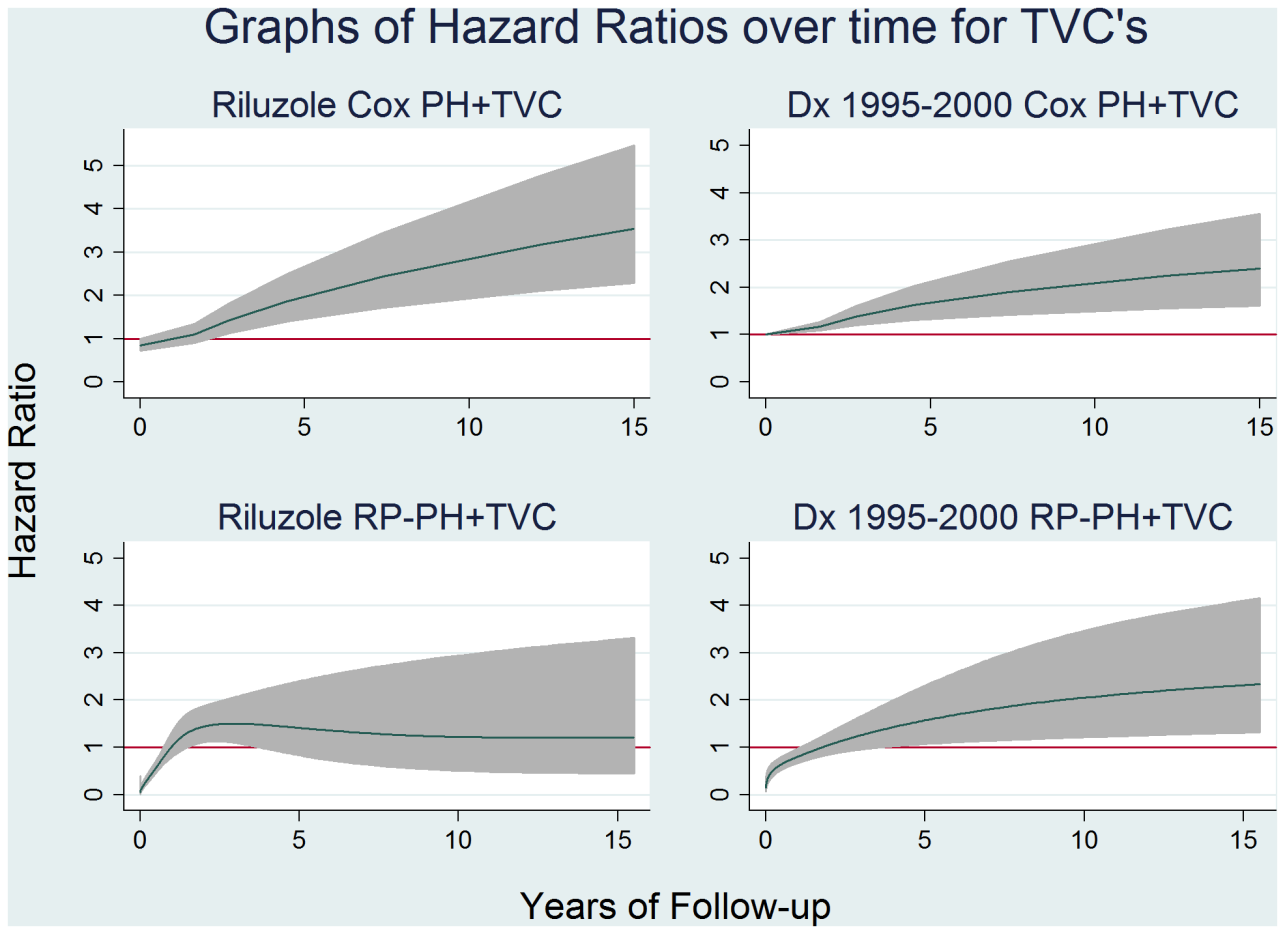
Graphs of time varying covariates. Note 1: The RP graph for riluzole is drawn with d.f. = 3 for time varying spline knots whilst the 1995–2000 graph is drawn with d.f. = 1 for time varying spline knots. These values were decided after comparison of AIC and BIC values of multiple possibilities. Note 2: While the group diagnosed between 2006–2010 also had P = 0.02 when modeled as a time varying covariate under Cox PH, the graph was unimpressive as it was limited to 5 years follow up and had 95% CI’s close to 1 at all points, and therefore has not been included.

The same time varying components were selected for both Cox PH and RP-PH models – riluzole and period of diagnosis. Hazard ratios versus time are shown in Figure 3. As can be seen we have graphed the 1995 to 2000 period instead of other periods. This is because on fitting the RP model, the *stpm2* command forced the use of dummy variables instead of a categorical variable. On doing this it became clear that the 1995 to 2000 period was in fact the time period in breach of PH. However as this was the base group in our Cox regression this was not evident in the Cox models. From table 1, we can see 73% of all patients with missing values were diagnosed between 1995 & 2000 and thus excluded from model building. This likely explains the failure of the PH assumption in the Cox model.

The HR’s for riluzole over time from both models were unexpected. The Cox PH+TVC graph (Figure 3) shows no significant association with improved survival at any time point and in fact an increasingly significant association with worse survival over time, whilst the RP-PH+TVC graph (Figure 3) shows association with improved survival only in the first year. Our Cox PH model with age as timescale (model 2) had failed to find a breach of PH assumption for riluzole (HR 0.74 95% CI: 0.63–0.88) – these combined results indicating probable residual confounding by age in model 1.

**Figure 4:**
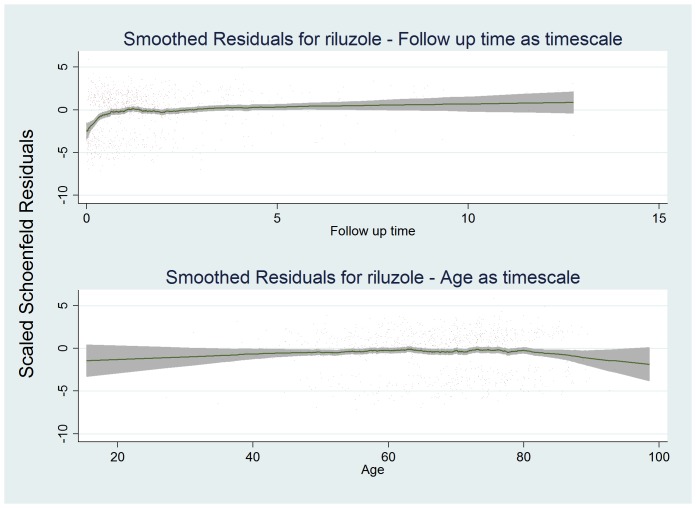
Smoothed Schoenfeld residuals from Cox PH models for riluzole use by years of followup. Nearest neighbour smoothed scaled Schoenfeld residuals for riluzole modeled with time of diagnosis as timescale origin (upper graph), and with age of diagnosis as timescale (lower graph). Deviation from linear trend can be seen in the first year on the upper graph. It is likely that this is caused by the greater power in detecting an effect of riluzole in older people due to the non-random distribution of riluzole use across age, combined with residual confounding by age - older people have poorer survival even if on riluzole. The combined effect leads to the appearance that riluzole is more effective in the first year (Figure 3), when in fact we have reduced power to detect the effect of riluzole in younger people (table 4), who are generally more likely to survive beyond one year (Figure 2). The lower graph using age as time scale origin does not show this trend and the PH assumption is not breached, however there is fluctuation dispersed over time. Note that a) the distribution of observations in time is altered as can be seen from the graph timescales and b) observations are reordered in time (not obvious from graph). Both features affect the evaluation of the proportional hazards assumption as Cox PH modeling is effectively a ranked method.

To better understand these results we graphed scaled Schoenfeld residuals from different timescales (Figure 4), performed stratified modeling by age group and examined crude associations of riluzole use by age strata (Table 4). The stratified HR’s demonstrate an age related effect of riluzole, however the CI’s narrowed with increasing age. The cross tabulation showed that riluzole usage rates correlate with age. Given this, and considering that model 2 showed no breach of PH, it seems likely the failure of the PH assumption for riluzole and apparently time varying nature of riluzole is due to a combination of residual confounding by age, and the observational nature of the study leading to a reduced power to detect the effect of riluzole in younger ages.

**Table 4:** Breakdown of Hazard ratios and usage rates of riluzole by age group.

a) Hazard ratios for riluzole stratified by age group after adjustment in multivariable Cox models					
Model	Cox PH			Cox PH	
Timescale	N	Time on study		Age	
		Riluzole HR (95% CI)*	P value	Riluzole HR (95% CI)**	P value
Age group					
25–54	218	0.74 (0.45–1.21)	0.235	0.83 (0.50–1.40)	0.489
55–64	309	0.74 (0.53–1.02)	0.067	0.82 (0.59–1.14)	0.244
65–74	356	0.73 (0.55–0.98)	**0.033**	0.78 (0.59–1.05)	0.099
75+	200	0.65 (0.46–0.93)	**0.019**	0.69 (0.48–0.98)	**0.040**
**b) Cross tabulation of riluzole use by age group**					
**Age group**	**N**	**Riluzole use:**	**No**	**Yes**	**Ratio (Y/N)**
25–54	218		32	186	5.81
55–64	309		60	249	4.15
65–74	356		115	241	2.10
75+	200		83	117	1.41

* Age stratified implementation of Model 1, table 3.

** Age stratified implementation of Model 2, table 3.

Table S1 shows a comparison of a Cox model (model 3) constructed using first date of diagnosis and then date of onset as study entry points. The date of onset model was constructed using diagnostic delay first as a linear variable (model S1) and then as a grouped variable (model S2) to illustrate the effect of correlation between diagnostic delay and survival time. This occurs when diagnostic delay is included as a linear term in a model with onset date as study entry, but is less of a concern when diagnostic delay is included as a grouped variable only (i.e. as a marker of slow, medium and fast disease progression).

## Discussion

On comparing different models there was no fully satisfactory model, however the Cox PH+TVC’s model was clearly inadequate. On balance we chose model 3, the RP-PH model as the best-fit model. Considering our analysis of TVC’s, it did not seem appropriate to include them in the best-fit model. It also did not seem prudent to accept the age as timescale models as superior since the acceptance of Cox PH despite missing values in earlier diagnostic years suggested over-fitting. On balance we prefer the Royston-Parmar model over Cox as it comes with the advantages of parametric models whilst closely matching the Cox estimates. The flexible nature of the RP models provided an excellent fit to non-parametric cumulative hazard and survival curves, enabled a greater understanding of breaches of the PH assumption and allowed us to plot smoothed survival curves for subgroups of the population. We found the Royston-Parmar models to provide great flexibility in particular for modeling time varying effects.

In keeping with the ALS literature [1], [3], [6], [14], [17] we found that bulbar onset ALS was significantly associated with poorer prognosis. The finding of significantly poorer prognosis for definite El Escorial disease is in keeping with several other studies [14], [17] and consensus opinion [3], although not all studies found El Escorial category to be associated with survival [15], [16]. Our findings that attendance at ALS clinic and riluzole use were associated with improved prognosis are consistent with the literature [1], [3], [5], [6], [17], although the effect of riluzole may be underestimated due to a likely lack of statistical power in younger people (Table 4). However, NIV use was associated with poorer prognosis when used in limb onset and generalized onset disease, but not in bulbar onset disease. Whilst this finding contrasts results from clinical trials [7], it is likely that without randomization, the NIV users in our study were a self-selecting group suffering a more severe disease course. Furthermore we have not accounted for known confounders such as NIV compliance [1], [13]. A puzzling finding of the initial Cox PH and RP-PH models was that of an apparently poorer prognosis in later years of the study. However the RP model including TVC’s allowed us to determine that the years 1995–2000 were in breach of proportional hazards. Therefore we believe that these findings are artefactual, and likely caused by a selection bias due to temporal concentration of missing values.

As a population based cohort analysis of incident cases with negligible loss to follow up (<1%) our study is of robust design. We have performed a detailed statistical analysis using multiple techniques that gave a deeper understanding of the data. We were also able to explore the use of age at diagnosis as a timescale – a technique of increasing popularity for controlling for confounding by age [18]–[20]. However after careful examination of our data, we found this method to obscure breaches of the PH assumption that are likely due to other causes (e.g. temporal concentration of missing data). Through this analysis we have shown the importance of investigating thoroughly breaches of the proportional hazards assumption.

Our study did suffer from some weaknesses including a number of patients excluded due to missing values (15%). Whilst we could have performed imputation of missing data, given the concentration of those missing values in earlier years that did not seem wise. Those missing values had several significant associations with key variables that we believe introduced a bias in the early years of the study. We have also demonstrated how the observational nature of our data may have reduced power to detect the effect of riluzole. Although the date of diagnosis does not hold specific meaning in the natural course of ALS, the date is significant as a marker for the commencement of interventions (particularly riluzole and ALS clinic attendance). As the hazard ratios of interventions were of primary interest to us, this prompted the decision to use diagnosis date to mark study entry. Use of onset date may be more appropriate in studies primarily interested in the survival association of clinical features present at onset (i.e. site of onset, Escorial category, etc). Such considerations are likely more important when numbers in subgroups are low.

In summary, median survival in Irish ALS patients diagnosed from 1995 to 2010 was 1.27 years from diagnosis and 2.39 years from symptom onset. The importance of traditionally recognized prognostic markers including age, bulbar onset disease, El-Escorial Definite ALS was confirmed. Riluzole use and attendance of ALS clinic were associated with improved prognosis. We found a linear relationship between diagnostic delay and improved survival. In addition we found Royston-Parmar flexible parametric modeling to be an excellent parametric alternative to Cox PH modeling. Testing of proportional hazards assumptions followed by thorough investigation of breaches proved invaluable in interpreting results and understanding deficiencies inherent to observational datasets.

## Supporting Information

Table S1 **Comparison of final preferred model using date of diagnosis and date of onset as timescale.** Note that model building was not performed for models S1 or S2 as would be appropriate for a formal survival analysis on the onset date timescale – instead the model specification of model 3 was implemented under onset date timescales as a tool to illustrate the effect of choice of timescale on hazard ratios. It should be noted that formal model building on the onset date timescale may result in inclusion of different model terms. The same patients were included in all models. Model S2 included diagnostic delay as a grouped categorical variable only to avoid the problem of correlation between diagnostic delay and overall survival that is of most concern when diagnostic delay is included as a linear effect under the onset date timescale as in model 2. In general fluctuations across the three models were low, although bulbar onset disease had a marginally higher HR in both model S1 & S2– thus appearing as a more significant negative hazard. Riluzole also had a higher HR in S1 & S2– however in this case it appeared as a less significant protective factor than in model 3. The HR for diagnostic delay was considerably lower in S1 compared to model 3– although due to the correlation issue it seems unwise to interpret this finding. The interaction term between NIV and general onset disease varied significantly between models – probably due to low numbers in this group (n = 10).
(DOCX)
