# Supplementary material for: Survival Analysis of Irish Amyotrophic Lateral Sclerosis Patients Diagnosed from 1995–2010
Source: PLoS One. 2013 Sep 30;8(9):e74733. doi: 10.1371/journal.pone.0074733 (PMC3786977; doi:10.1371/journal.pone.0074733)
Supplement: Table S1 — Comparison of final preferred model using date of diagnosis and date of onset as timescale. Note that model building was not performed for models S1 or S2 as would be appropriate for a formal survival analysis on the onset date timescale – instead the model specification of model 3 was implemented under onset date timescales as a tool to illustrate the effect of choice of timescale on hazard ratios. It should be noted that formal model building on the onset date timescale may result in inclusion of different model terms. The same patients were included in all models. Model S2 included diagnostic delay as a grouped categorical variable only to avoid the problem of correlation between diagnostic delay and overall survival that is of most concern when diagnostic delay is included as a linear effect under the onset date timescale as in model 2. In general fluctuations across the three models were low, although bulbar onset disease had a marginally higher HR in both model S1 & S2– thus appearing as a more significant negative hazard. Riluzole also had a higher HR in S1 & S2– however in this case it appeared as a less significant protective factor than in model 3. The HR for diagnostic delay was considerably lower in S1 compared to model 3– although due to the correlation issue it seems unwise to interpret this finding. The interaction term between NIV and general onset disease varied significantly between models – probably due to low numbers in this group (n = 10). (DOCX) [file pone.0074733.s001.docx]

Table S1 Comparison of final preferred model using date of diagnosis and date of onset as timescale

| Model Number |  | **Model 3** | **Model S1** | **Model S2** |
| --- | --- | --- | --- | --- |
| Model Type |  | **Royston-Parmar** | **Royston-Parmar** | **Royston-Parmar** |
| Model scale |  | **PH (df 3)** | **PH (df 3)** | **PH (df 3)** |
| Model Entry time |  | Diagnosis date | Onset date | Onset date |
| Variable | Strata | HR (95%CI) | HR (95%CI) | HR (95%CI) |
| Age dx | Per 10 yrs increase | 1.34 (1.26 – 1.42) | 1.34 (1.26 – 1.42) | 1.34 (1.26 – 1.42) |
| El Escorial | Possible | 1 | 1 | 1 |
|  | Suspected | 0.96 (0.50 – 1.84) | 1.01 (0.53 – 1.95) | 0.88 (0.46 – 1.69) |
|  | Probable | 1.30 (1.03 – 1.64) | 1.23 (0.98 – 1.55) | 1.34 (1.06 – 1.68) |
|  | Definite | 1.47 (1.17 – 1.84) | 1.41 (1.13 – 1.76) | 1.41 (1.13 – 1.76) |
| Site of onset (no NIV) | Limb onset | 1 | 1 | 1 |
|  | Bulbar onset | 1.58 (1.33 – 1.87) | 1.75 (1.48 – 2.08) | 1.78 (1.51 – 2.11) |
|  | General onset | 1.17 (0.83 – 1.65) | 1.21 (0.86 – 1.71) | 1.14 (0.81 – 1.61) |
| Diagnostic Delay | Per 12 weeks delay | 0.96 (0.94 – 0.97) | 0.88 (0.86 – 0.90) | - |
|  | <31 wks | - | - | 1 |
|  | 31 – 55 wks | - | - | 0.59 (0.51 – 0.70) |
|  | > 55 wks | - | - | 0.27 (0.22 – 0.32) |
| Year of diagnosis | 1995 – 2000 | 1 | 1 | 1 |
|  | 2001 – 2005 | 1.26 (1.06 – 1.51) | 1.21 (1.01 – 1.44) | 1.19 (1.00 – 1.42) |
|  | 2006 – 2010 | 1.15 (0.96 – 1.39) | 1.18 (0.98 – 1.42) | 1.16 (0.96 – 1.40) |
| Attend ALS clinic | Yes | 0.74 (0.64 – 0.86) | 0.74 (0.64 – 0.86) | 0.71 (0.61 – 0.82) |
| Riluzole | Yes | 0.72 (0.61 – 0.85) | 0.77 (0.65 – 0.91) | 0.80 (0.68 – 0.94) |
| NIV usage | NIV + Limb onset | 1.49 (1.21 – 1.84) | 1.47 (1.20 – 1.81 | 1.62 (1.32 – 1.99) |
|  | NIV + Bulbar onset | 1.09 (0.83 – 1.44) | 1.25 (0.95 – 1.66) | 1.23 (0.93 – 1.63) |
|  | NIV + General onset | 2.74 (1.31 – 5.71) | 3.45 (1.68 – 7.10) | 1.71 (0.83 – 3.54) |
